# Modulated Electro-Hyperthermia Supports the Effect of Gemcitabine Both in Sensitive and Resistant Pancreas Adenocarcinoma Cell Lines

**DOI:** 10.3389/pore.2021.1610048

**Published:** 2021-12-10

**Authors:** Gertrud Forika, Eva Kiss, Gabor Petovari, Titanilla Danko, Aron Bertram Gellert, Tibor Krenacs

**Affiliations:** ^1^ 1st Department of Pathology and Experimental Cancer Research, Semmelweis University, Budapest, Hungary; ^2^ 1st Department of Internal Medicine and Oncology, Oncology Profile, Semmelweis University, Budapest, Hungary; ^3^ Department of Thoracic Surgery, National Institute of Oncology and Semmelweis University, Budapest, Hungary

**Keywords:** apoptosis, cell cycle arrest, pancreas adenocarcinoma, modulated electro-hyperthermia, gemcitabine resistance, combined treatment

## Abstract

The poor prognosis of pancreatic ductal adenocarcinoma (PDAC) is frequently associated to high treatment resistance. Gemcitabine (GEM) alone or in combination is the most used chemotherapy for unresecable PDACs. Here we studied whether modulated electro-hyperthermia (mEHT), a non-invasive complementary treatment, can support the effect of GEM on PDAC cells *in vitro*. The LD20 for the GEM-resistant Panc1 cells proved to be 200× higher than for the drug-sensitive Capan1. The mEHT alone caused significant apoptosis in Capan1 cultures as confirmed by the elevated SubG1 phase cell fraction and increased number of cleaved Caspase-3 positive cells 48 h after treatment, with an additive effect when GEM was used after hyperthermia. These were accompanied by reduced number of G1, S, and G2/M phase cells and elevated expression of the cyclin-dependent kinase inhibitor p21^waf1^ protein. In GEM-resistant Panc1 cells, an initial apoptosis was detected by flow cytometry 24 h after mEHT ± GEM treatment, which however diminished by 48 h at persistent number of cleaved Caspase-3 positive tumor cells. Though GEM monotherapy reduced the number of tumor progenitor colonies in Capan1 cell line, an additive colony inhibitory effect of mEHT was observed after mEHT + GEM treatment. The heat shock induced Hsp27 and Hsp70 proteins, which are known to sensitize PDAC cells to GEM were upregulated in both Capan1 and Panc1 cells 24 h after mEHT treatment. The level of E-Cadherin, a cell adhesion molecule, increased in Capan1 cells after mEHT + GEM treatment. In conclusion, in GEM-sensitive PDAC cells mEHT treatment alone induced cell death and cell cycle inhibition and improved GEM efficiency in combination, which effects were milder and short-term up to 24 h in the GEM-resistant Panc1 cells. Our data further support the inclusion of hyperthermia, in particular of mEHT, into the traditional oncotherapy regimens of PDAC.

## Introduction

Pancreatic cancer is one of the most fatal malignant tumors worldwide. Pancreatic ductal adenocarcinomas (PDAC), representing more than 90% of all pancreatic cancers are characterized by high aggressiveness and mortality rates, and have limited response to available anticancer treatments [1, 2]. The unspecific symptomatology and consequently late diagnosis lead to an only 10% 5-years survival rate [3]. Gemcitabine (GEM) alone or in combination with platinum or the FOLFIRINOX regimen are the most used chemotherapies for unresecable PDACs. However, the success of these treatments has been still modest with a median survival of 6.8 months for GEM monotherapy and 11.1 months in combination with FOLFIRINOX [4]. Therefore, we tested a complementary non-invasive electro-hyperthermia treatment to improve gemcitabine efficiency in PDAC.

Gemcitabine is a nucleoside analog (2′,2′-difluoro-2′-deoxycytidine) and is preferred for patients who are not suitable for aggressive chemotherapy [5], although the high GEM resistance of PDAC is well known [6]. As a prodrug, GEM traverses cell membranes *via* nucleoside transporters and it is activated by cytoplasmic deoxycytidine kinase into gemcitabine di- and triphosphate.

Hyperthermia has gained increasing popularity in cancer treatment to complement traditional and even targeted therapies. Combining with radio- or chemotherapy it can be effective against several cancer types [7]. In cell cultures, hyperthermia can induce cytotoxicity especially in the M-phase of tumor cells, which can synergize with or cause an additive tumor cell killing effect when it is combined with platinum or antimetabolites [8].

Modulated electro-hyperthermia (mEHT) is a loco-regional hyperthermia which uses radiofrequency to generate selective heat in malignant tissues as a result of the metabolic shift towards glycolysis in cancer cells even under oxidative conditions, known as the Warburg effect [9, 10]. This results in elevated lactate and ion concentration, as well as increased electric permittivity, compared to the surrounding normal tissues [11]. Selective heat may induce heat and cell stress in tumors which can lead to cancer cell death. Many clinical trials and retrospective studies confirmed improved overall response and higher response rate when chemo- or radiotherapy was combined with mEHT [12–16]. However, the molecular changes caused by mEHT treatment have only been partly identified and may show some diversity depending on the inherent genetic background of the treated cancers. We have recently reviewed the mEHT-induced molecular changes in cancer models [17]. Accordingly, several pathways have been associated with mEHT-related programmed cell death response, including the exhaustion of chaperones such as heat shock proteins, the promotion of DNA strand breaks, and the upregulation and release of damage-associated molecular patterns (DAMP). Modulated electro-hyperthermia can induce either caspase-dependent or independent apoptosis which was demonstrated in colorectal carcinoma (CRC) models [18]. In another study, mEHT treatment caused high release of mitochondrial Cytochrome C in TP53 wild CRC *in vitro* which was accompanied with the cleavage and activation of both Caspase-8 and Caspase-3 proteins [19]. However, in a TP53-mutant CRC model the major programmed cells death response was caspase-independent with the upregulation and activation of apoptosis inducing factor (AIF) [20].

In this study, GEM sensitive (Capan1) and GEM resistant (Panc1) PDAC cell lines were tested using GEM either alone or in combination with standard mEHT to see whether hyperthermia can improve treatment efficacy and if so which molecular pathways are involved in this effect. We also took an effort to develop GEM-resistant cell lines to see if mEHT treatment could still be efficient on tumor clones selected out by the earlier administration of the drug.

## Materials and Methods

### Cell Culture Conditions

Pancreatic adenocarcinoma cell lines, Capan1 (CLS Cell Lines Service GmbH, Eppelheim, Germany) and Panc1 (ATCC, Teddington, Middlesex, United Kingdom) were grown in Iscove’s Modified Dulbecco’s Medium and Dulbecco’s Modified Eagle Medium (IMDM LM-I1092 and DMEM LM-D1111, Biosera, Boussens, France), respectively; enriched with 10% heat inactivated fetal bovine serum (FBS FB-1001H, Biosera, Boussens, France) and 80 mg Genta-Gobens (Gentamicin, Laboratorios Normon, Madrid, Spain). In case of Capan1, the media were also supplemented with 1% 2 mM L-glutamine (XC-T1715 Biosera, Boussens, France). Cells were kept in a humidified incubator at 37°C with an atmosphere containing 5% CO_2_.

For treatments, we harvested subconfluent cell cultures using 0.25% trypsin and 0.22 mg/ml ethylenediaminetetraacetic acid (Trypsin-EDTA, XC-T1717/100, Biosera, Boussens, France). After centrifugation (300 g, 5 min), the total number of treated cells were determined.

### Resistant Cell Lines

GEM-resistant cell lines were established using cell selection method [21]. Briefly, cells were exposed to increasing concentrations of GEM starting from 0.05 nM for Capan1 and 1 nM for Panc1. After each dose step, cells were cultured until a subconfluent population was reached. Unfortunately, when the concentration reached 3.2 nM GEM for Capan1 and 16 nM GEM for Panc1, most cells were detached and died in both cultures. Two more attempts were made where cells were subcultured repeatedly at lower GEM concentrations (0.8 nM for Capan1 and 8 nM for Panc1). Despite, after a few-week continuous passaging, cell death was observed again.

### Hyperthermia and Gemcitabine Treatment

For mEHT treatment, Lab-EHY 200 device (Oncotherm Kft, Budaors, Hungary) was used with all accessories customized for cell suspension treatment. The treatment bag containing 1.5 ml of 10^6^ cells/ml suspension was submerged into the treatment cuvette filled with distilled water. One temperature sensor was inserted directly inside the treatment bag and another one into the surrounding water. The treatments lasted for 65 min including 5–10 min preheating period at 10 W average input power, and 55–60 min maintaining period using 2.4 W average input power. The amplitude modulated electric field generated 42 ± 0.3°C inside the treatment bag.

For gemcitabine (Fresenius Kabi Oncology Plc., Hampshire, United States) treatment, a 1,000 µM stock solution was made in 0.1 M neutral phosphate-buffered saline (PBS) buffer. This stock solution was diluted in cell culture media to achieve the preferred concentration and added to the cells, or added right after the 60 min mEHT treatment when combined. Samples were collected and tested at 0, 24, 48 or 72 h from the start of any treatment. In the 60 min mEHT monotherapy group tumor cells were grown in normal culture media, until sampling. In the GEM treatment groups cells were grown in GEM containing media either from the beginning, or after 60 min mEHT, when the two treatments were combined (mEHT + GEM), for the rest of time.

### Analysis of Cell Morphology and Immunocytochemistry

To analyze cell morphology, one piece of 18 × 18 mm sterilized histology glass coverslips was placed into each well of a 6-well plate. We seeded 2 × 10^5^ cells/2 ml of Capan1 or 10^5^ cell/2 ml of Panc1 into wells supplemented for 24 and 48 h, with 0.5 nM GEM in Capan1 cultures and 12 nM or 100 nM GEM for Panc1. Hematoxylin-eosin staining was performed. Cells were washed twice with PBS then fixed in 4% formaldehyde solution at room temperature. After washing the coverslips with distilled water, cells were stained with hematoxylin (2 min) and eosin (2 min). Finally, running tap water was used for bluing the samples.

For immunocytochemistry, after formalin fixation, we performed a 30 min peroxide block with 3% hydrogen peroxide diluted in methanol. Cell membranes were permeabilized with TBST buffer for 30 min, made up of 0.01 mol/L Tris-buffered saline pH 7.4 (TBS) containing 0.5% Tween-20 (Fisher Scientific United Kingdom Ltd., Loughborough, United Kingdom). Nonspecific protein blocking was made in 3% bovine serum albumin (Probumin, BSA, 82-100-6, Merk, Darmstadt, Germany) for 30 min. The primary antibodies were diluted in 1% BSA, the secondary antibodies were diluted in TBST. Primary antibodies such as rabbit monoclonal cleaved Caspase-3 (1:300, clone: 5A1E, #9664, Cell Signaling, Danvers, MA, United States), rabbit monoclonal Cytochrome C (1:100, clone: 136F3, #4280S, Cell Signaling), rabbit monoclonal E-cadherin (1:100, clone: EP700Y, #246R-14, Cell Marque, Rocklin, CA, United States) and mouse monoclonal N-Cadherin (1:100, clone: 32/N-Cadherin from BD Bioscience, NJ, United States) were incubated for 2 h at room temperature. After rigorous washing, polymer-peroxidase labeled mouse or rabbit IgG (Histols MR-T, Histopathology Ltd., Pecs, Hungary) was used for 1 h at room temperature, in case of N-Cadherin this step was preceded by a 30-min incubation with signal enhancer (Histols MR-T, Histopathology Ltd.). The chromogen reaction was revealed using a DAB chromogen/hydrogen peroxide kit (DAB Quanto, TA-060QHDX, Thermo-Fisher, Cheshire, United Kingdom). Cell nuclei were counterstained with hematoxylin. All stained coverslip cultures were dehydrated, mounted onto glass slides, and digitalized (Pannoramic scanner, 3DHISTECH, Budapest, Hungary). The cleaved Caspase-3 slides were analyzed using the QuantCenter image analysis software package (3DHISTECH).

### Cell Viability Assay With Resazurin

Control and mEHT treated cells were seeded in a 96-well plate, at a concentration of 10^4^ cells/well in 200 μl cell culture media. Culture media were supplemented with gemcitabine at different concentrations between 0 and 100 µM and incubated for 24, 48, and 72 h.

Resazurin is a cell-permeable redox-sensitive dye, which is reduced to resorufin by aerobic respiratory enzymes within cells. In contrast to resazurin, resorufin fluoresces when exposed to green light, thereby it is widely used to measure the viability of cells. Resazurin sodium salt (R7017 Sigma Aldrich, St. Louis, MO, United States) was diluted in PBS to make a stock solution of 0.3 mg/ml concentration which was further diluted in 1:10 when added directly into cell culture media. The measurement was done using Fluoroskan FL Microplate Fluorometer (5200110 Thermo-Fisher, Cheshire, United Kingdom) at excitation and emission wavelength of 570/590 after 2 h incubation of resazurin.

### Flow Cytometry Measurement of Apoptosis/Cell Cycle

The preparation of cells for flow cytometry was executed as we described before with minor changes [22]. Briefly, cells were released from the bottom of six well plates by trypsinization and were collected including their supernatants in Eppendorf tubes. After washing in PBS twice and centrifugation at 300 × g for 4 min, cells were fixed in ice-cold ethanol overnight for cell cycle measurement or stained without fixation for apoptosis analysis. For apoptosis measurement, cells were stained with Alexa Fluor 647 Annexin V (1:100; #640912, BioLegend, San Diego, CA, United States) and 1 µl of 1 mg/ml stock solution (diluted in PBS) of propidium iodide (PI, 1304MP, Thermo-Fisher, Cheshire, United Kingdom) in 100 µl of Annexin V binding buffer (#422201 BioLegend, San Diego, CA, United States). After a 15-min incubation at room temperature, we carried out the direct detection of apoptotic and necrotic bodies. The cell cycle analysis was performed on the next day after the cells were washed twice with PBS and incubated with 20 ng RNaseA (R5503, Sigma-Aldrich, Inc., St. Louis, MO, United States) and 10 µl of 1 mg/ml PI in 250 µl PBS at 4°C for 60 min.

Flow cytometry was performed on a CytoFLEX Flow Cytometer using CytExpert software (Beckman Coulter, Indianapolis, IN, United States).

### Colony Forming Assay

Clonogenic assay was performed as we described before [22] with minor changes: 10^3^ Panc1 and 10^4^ Capan1 cells were seeded in each well of a 6-well plate in 2 ml cell specific medium or in medium supplemented with the corresponding concentration of GEM. After 48 h, the media were removed from each well and replaced by fresh cell culture media without any drug added. The cells were cultured for another 14 days, then fixed in 4% formaldehyde at room temperature. After staining with 0.1% crystal violet solution, the colonies consist of more than 50 cells were manually counted.

### Capillary Western Blot

Western blot analysis was performed as we described before [22]: Capan1 and Panc1 cells were seeded at 2 × 10^5^ and 10^5^ cells/2 ml respectively, in each well of a 6-well plate after mEHT treatment and GEM treatment for 24 and 48 h. After removing the media and washing the cells with PBS twice, the proteins were extracted with 50 µl/well freshly prepared extraction buffer: 20 mM Tris, 2 mM EDTA, 150 mM NaCl, 1% Triton X-100; supplemented with 10 mM NaF, 0.5 mM NaVO_3_, and 1:200 Protease Inhibitor Cocktail (P8340, Sigma-Aldrich, St. Louis, MO, United States). Cell lysate was incubated for 30 min on ice, then centrifuged at 4°C and 12,000 rpm for 20 min. The total protein content was quantified by the Bradford reagent (#500-0205, BioRad, Hercules, CA, United States) and the samples were stored at −20°C until WES Simple analysis.

WES capillary Western blot device (ProteinSimple, San Jose, CA, United States) was used with the 12–230 kDa Jess/Wes separation module kit (SM-W004) according to the manufacturer’s instructions. The kit included a 25-capillary cartridge (12–230 kDa), pre-filled microplates with running buffer, wash buffer, 10× sample buffer, and an EZ standard pack with a 12–230 biotinylated ladder, a fluorescent 5× master mix, and a dithiothreitol (DTT) containing tube. The lyophilized DTT and biotinylated ladder were suspended in the right amount of distilled water. The fluorescent 5× master mix was suspended in 20 µl previously prepared DTT solution and 20 µl 10× Sample Buffer. The samples were diluted to a concentration of 1 µg/µl in 100× diluted “10× Sample Buffer,” five parts of sample solution and one part of fluorescent 5× master mix were mixed together, then the mixture was heated for 5 min at 95°C. The following primary antibodies were used: rabbit polyclonal BAX (1:30, #HPA027878, Sigma-Aldrich, St. Louis, MO, United States), mouse monoclonal p21^waf1^ (1:35, clone:70/Cip1/WAF1, #610234, BD Bioscience NJ 07417, United States), rabbit monoclonal Hsp27 (1:70, clone: D6W5V, #96357, Cell Signaling, Danvers, MA, United States), rabbit polyclonal Hsp70 (1:70, #4872, Cell Signaling, Danvers, MA, United States), rabbit monoclonal E-Cadherin (1:70, clone:EP700Y, #RM-2100, Thermo-Fisher, Cheshire, United Kingdom) and mouse monoclonal N-Cadherin (1:70, clone: 32/N-Cadherin, BD Bioscience NJ 07417, United States). We used peroxidase conjugated anti-rabbit and anti-mouse detection module reagents (ProteinSimple DM-001 and DM-002), and chemiluminescent substrate. The plates with all components were centrifuged at 2,500 rpm for 5 min, then the cartridges were inserted into the instrument which was used with the default settings of the software: stacking and separation at 395 V for 30 min; blocking for 5 min, primary and secondary antibodies both for 30 min; luminol/peroxide chemiluminescence detection for 15 min (exposure times were between 1 and 512 s). The electropherograms were checked then the automatic peak detection was manually corrected if it was required.

### Statistics

The experiments were carried out in minimum triplicate for each analysis. For statistical analysis we used nonparametric Kruskal-Wallis test, Dunn’s multiple comparison post-hoc test or nonparametric multiple T test integrated test calculated by GraphPad Prism software package (San Diego, CA, United States). Statistical significance was considered at *p* < 0.05 at a CI = 95%.

Each column in the graphs shows the mean and SD of the results gained from minimum three independent experiments including one or more parallel samples per group.

## Results

### Testing Gemcitabine Cytotoxicity Either Alone or in Combination With Hyperthermia

Both Capan1 and Panc1 PDAC cell lines are known to accumulate several tumor driver mutations resulting in a basic gemcitabine resistance, in particular for Panc1 [23, 24]. Unfortunately, our efforts to increase this resistance by using the multiple selection methods to support the potential effect of mEHT in combined therapy has failed.

**FIGURE 1:**
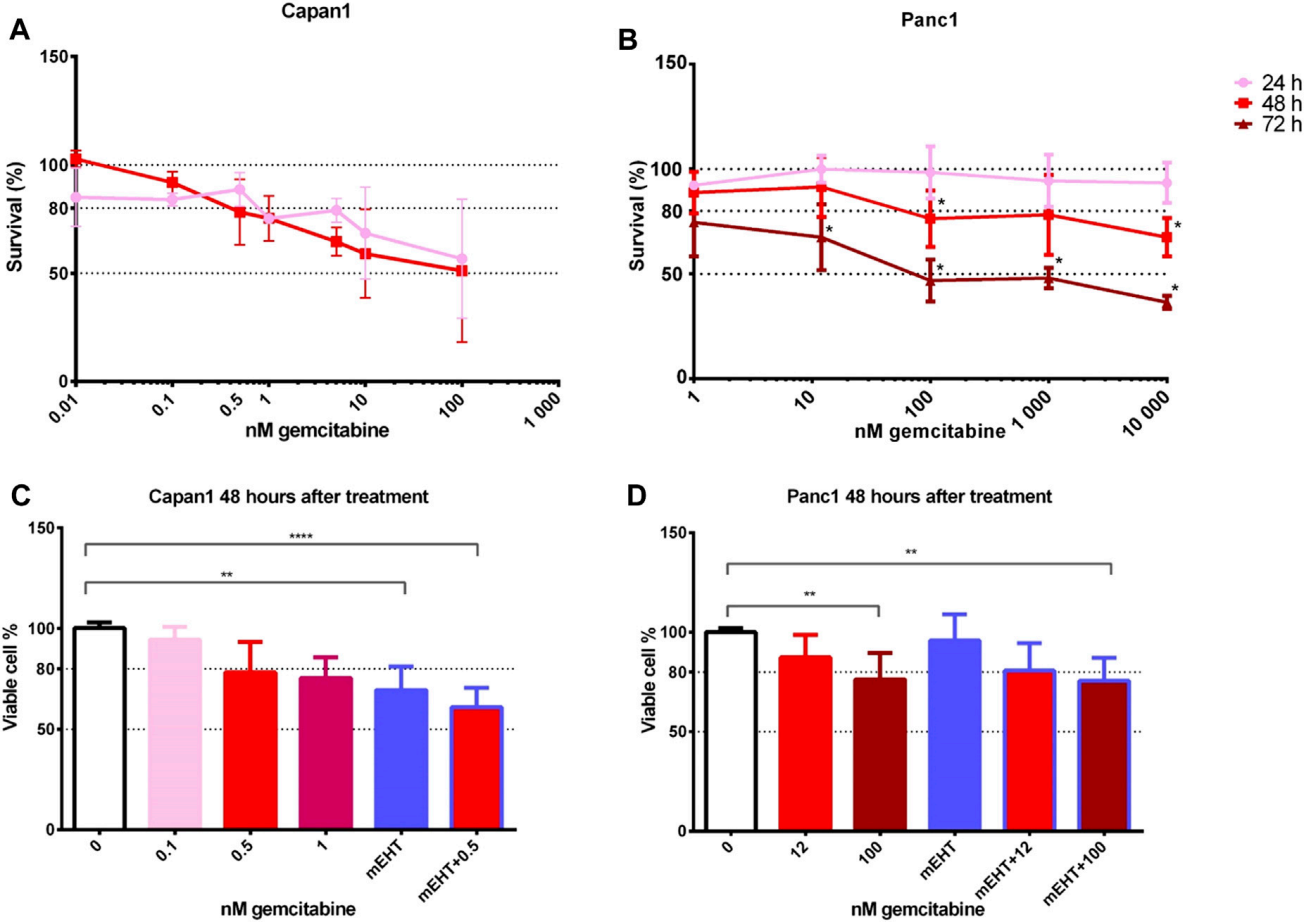
Cell viability after GEM treatment of Capan1 and Panc1 cell lines using resazurin assay **(A,B)**. The drug concentration required for an LD20 was 200-times higher for Panc1 than for Capan1 at 48 h (Capan1 LD20 = 0.5 nM GEM and Panc1 LD20 = 100 nM GEM) (*p < 0.05). Kruskal-Wallis statistical analysis of GEM, mEHT and mEHT + GEM treatments 48 h after mEHT treatment **(C,D)**. Sixty minutes of mEHT was effective both alone (*p* = 0.0013) and in combination with GEM in Capan1 cells (*p* < 0.0001). In Panc1 cell line, 100 nM GEM both alone and in combination with mEHT resulted in significant loss of tumor viability (*p* = 0.0097 and *p* = 0.0084, respectively).

Therefore, we tested gemcitabine cytotoxicity in the original Capan1 and Panc1 cell lines. We used gemcitabine in concentrations between 0–10,000 nM and cytotoxicity was measured by resazurin assay after 24, 48, and 72 h treatment (Figures 1A,B). The time-dependency of the GEM effect was reflected by the remarkable cell viability loss in both cell lines particularly at 48 and 72 h after treatment.

To analyze the potential complementary effect of mEHT on GEM treatment, cell cultures were treated with mEHT for 60 min then we added GEM for 48 h. This standard mEHT treatment was applied in all relevant experiments in this study. Drug concentrations were selected based on the cytotoxicity curves: 0.5 nM for Capan1 and 12 nM or 100 nM GEM for Panc1 cells (Figures 1C,D). In case of Capan1 cell line, mEHT treatment resulted in significantly reduced tumor cell viability with and without GEM. In Panc1 culture, 100 nM GEM treatment alone and combined with mEHT induced significant cell viability loss.

### Morphology and Mechanism of Treatment-Related Cell Death

**FIGURE 2:**
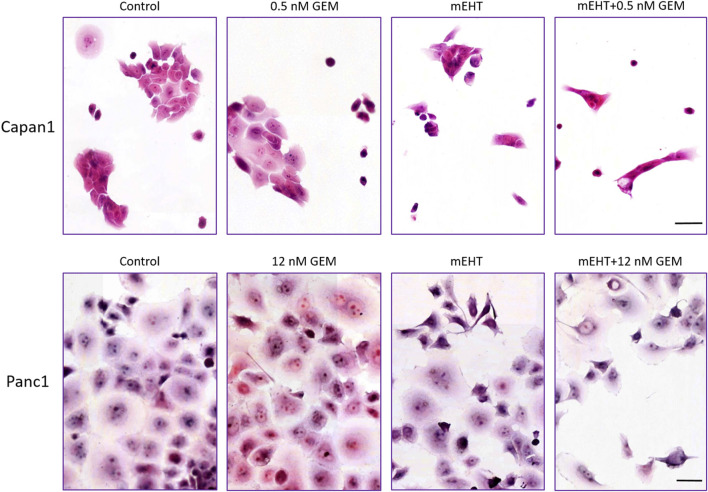
Hematoxylin-eosin staining 48 h post treatment. The formalin-fixed cells showed signs of apoptosis like karyopyknosis and karyorrhexis aside other morphological changes: hyperchromic nuclei, elongated cell shape, spatial tapering morphology, and disruption of interactions between the cells. Scale bar = 50 µm.

Panc1 cultures formed large sheets of adherent tumor cells as opposed to small cells groups of Capan1 cells. Treatments caused the detachment of damaged cells resulting in a decreased number of surviving adherent cell populations. Nevertheless, the morphological signs of cell death were visible from 1 day after treatments, particularly in the more adhesive Panc1 cultures (data not shown). H&E staining of the remaining adherent cells 48 h after treatment revealed obvious signs of apoptosis including elevated numbers of pyknotic tumor cells with chromatin condensation, high basophilia, and membrane blebbing (Figure 2). The most extensive cell detachment was observed in the double-treated and mEHT treated Capan1 cells. The signs and tendency of damage and cell loss were similar in Panc1 cultures but less obvious. GEM treatment primarily caused detachment of the cells with fewer apoptotic signs.

**FIGURE 3:**
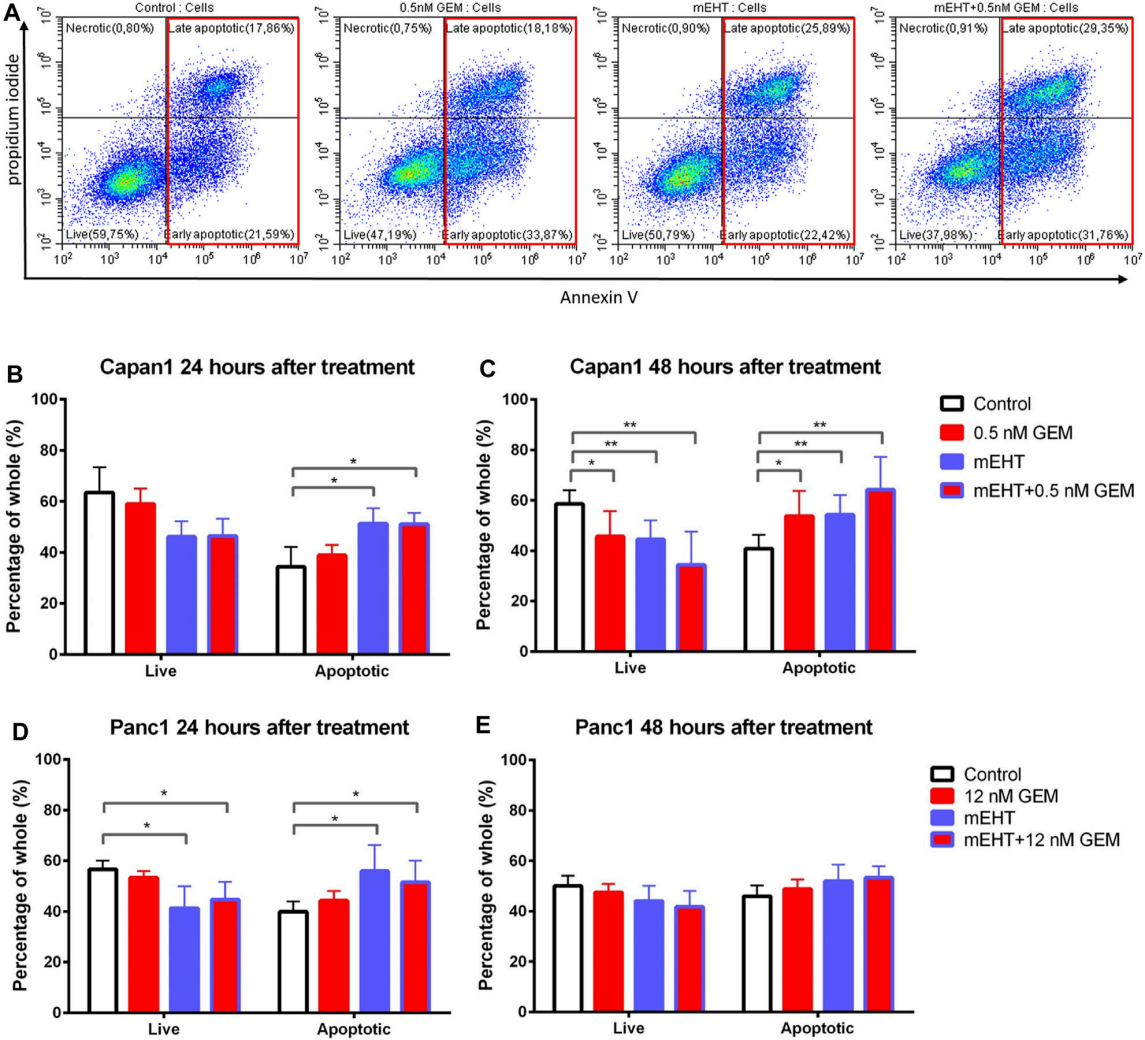
Flow cytometry of Capan1 and Panc1 cell lines labeled with PI and Annexin V at 24 and 48 h after treatment. Representative flow cytometry analysis of Capan1 cells 48 h after treatment. The red boxes represent apoptotic cell fractions **(A)**. The multiple T test showed a significant increase of apoptotic tumor cells in both Capan1 and Panc1 cells 24 h posttreatment in groups that were treated with mEHT alone or in combination with GEM compared to the control (*represents *p* < 0.05) **(B, D)**. Comparing the effect of 48 h treatments, significant alteration was detected only in case of Capan1 cells. The apoptosis increased after every treated group compared to the control with strong significance in case of groups that received mEHT alone or in combination (*represent *p* < 0.05 and ** represent *p* < 0.01) **(C,E)**.

To examine the mechanism of cell death, we prepared double-stained tumor cells after 24 and 48 h treatments using Annexin V and propidium iodide (PI). In flow cytometry measurements, those cells were considered as living cells which did not express any fluorescence signal, and apoptotic cells were determined that showed Annexin V with or without PI positivity (Figure 3A).

24 h after treatment, mEHT alone and in combination with GEM the apoptotic subpopulation was significantly elevated compared to the control in both cell lines (*p* < 0.05). In line with this, the number of live cells was decreased in these groups compared to the control, but the difference was significant only in Panc1 cultures (*p* < 0.05) (Figures 3B,D). 48 h after treatment significantly fewer live Capan1 tumor cells and significantly elevated apoptotic cells were detected in all three treated groups compared to the controls. In Panc1 cultures, the difference observed at 24 h was vanished by 48 h (Figures 3C,E).

### Treatment-Related Caspase-Dependent Apoptosis

To further clarify the pathways of apoptosis after treatments, Cytochrome C, BAX, and Caspase-8 protein levels were measured using Wes Simple analysis. The proportion of cleaved Caspase-3 positive cell fraction was determined by immunocytochemistry.

**FIGURE 4:**
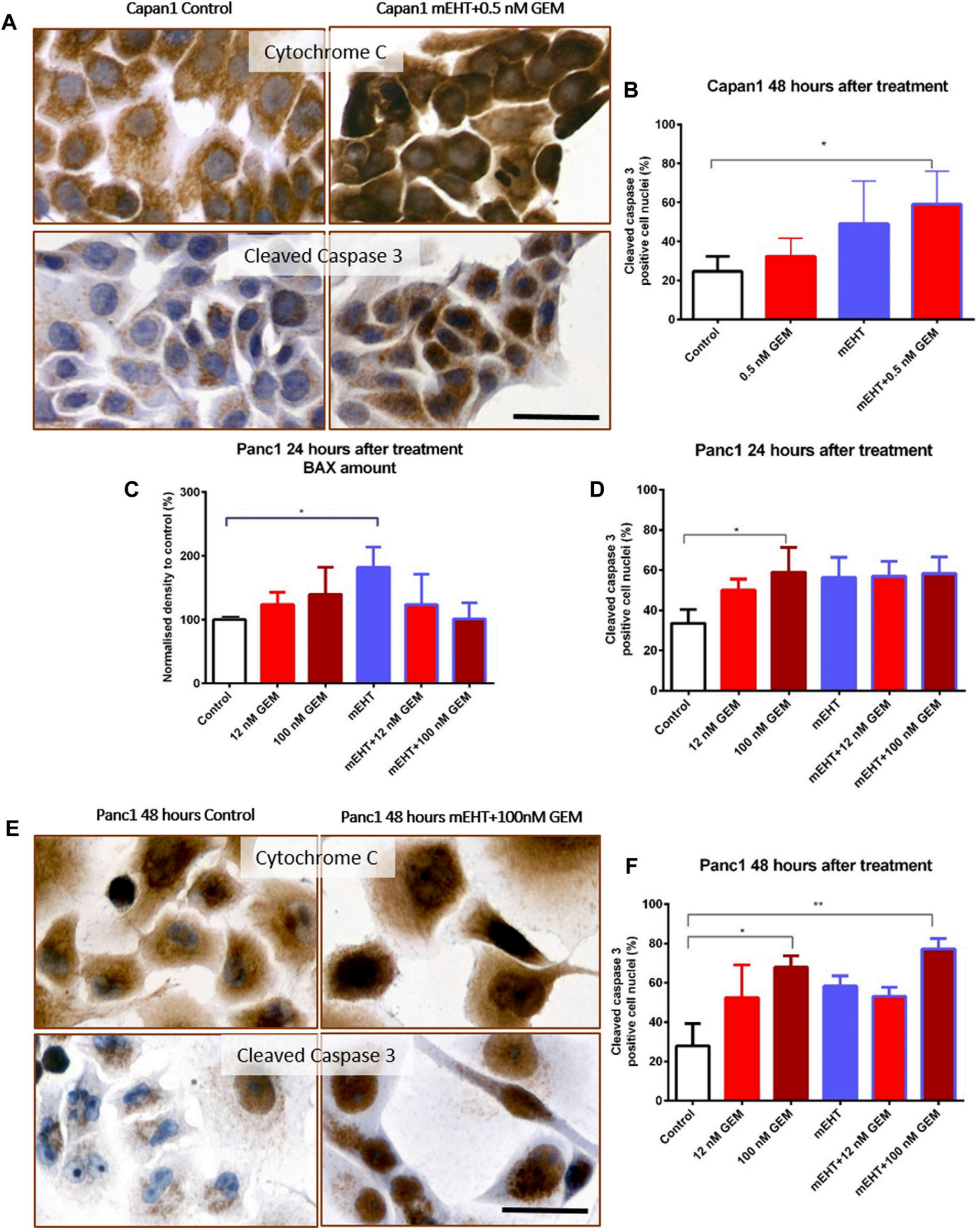
Immunocytochemistry of Cytochrome C and cleaved Caspase-3. The granular Cytochrome C became diffuse cytoplasmic in both cell lines after treatment as a sign of mitochondrial release **(A,E)**. Significant increase of nuclear cleaved Caspase-3 positivity was seen in the double-treated Capan1 cells compared to the control, *p* = 0.046 **(A,B)**. In Panc1 cells, the pro apoptotic BAX protein level was significantly elevated 24 h after mEHT treatment, and the number of cleaved Caspase-3 positive cells also increased in the 100 nM GEM treated group (*p* = 0.029 and *p* = 0.048) **(C,D)**. High cleaved Caspase-3 levels were maintained 48 h after 100 nM GEM treatment (*p* = 0.028) with the same elevation seen in mEHT + 100 nM GEM treated group (*p* = 0.0013) **(E,F)**. The scale bar represents 40 µm.

In Capan1 cell cultures 48 h after treatment, the Kruskal-Wallis test showed a statistically significant difference in the number of nuclear cleaved Caspase-3 positive tumor cells (Figures 4A,B) between the treated groups with *p* = 0.02. The post hoc test showed a significant elevation in the combined mEHT + GEM group compared to the control (*p* = 0.046). In Panc1 cultures, the nuclear cleaved Caspase-3 positive cell population also showed a significant elevation after 100 nM GEM treatment compared to the control at 24 (*p* = 0.048) and 48 h (*p* = 0.028). Similar results were observed in mEHT + 100 nM GEM treatment at 48 h (*p* = 0.013) (Figures 4D–F).

Western blot analysis of extrinsic and intrinsic apoptosis pathways showed no statistical difference either in Caspase-8 or in Cytochrome C levels between the treated and the control groups (data not shown). In Panc1 cells, BAX levels were significantly elevated 24 h after mEHT treatment (Figure 4C), which disappeared by 48 h (data not shown). The level of BAX did not show significant elevation after combined treatment either at 24 h or at 48 h. Since we presumed that the cytoplasmic translocation of Cytochrome C is not reflected by its change at protein level, immunocytochemistry was also performed. Indeed, the granular mitochondrial localization frequently became diffuse cytoplasmic in the treated cultures (Figures 4A,E), reflecting the potential release of the protein from the mitochondria and indirectly proving the contribution of BAX to mitochondrial damage.

### Combined Treatment-Related Cell Cycle Arrest and Reduced Post-G1 Phase Cell Fractions

**FIGURE 5:**
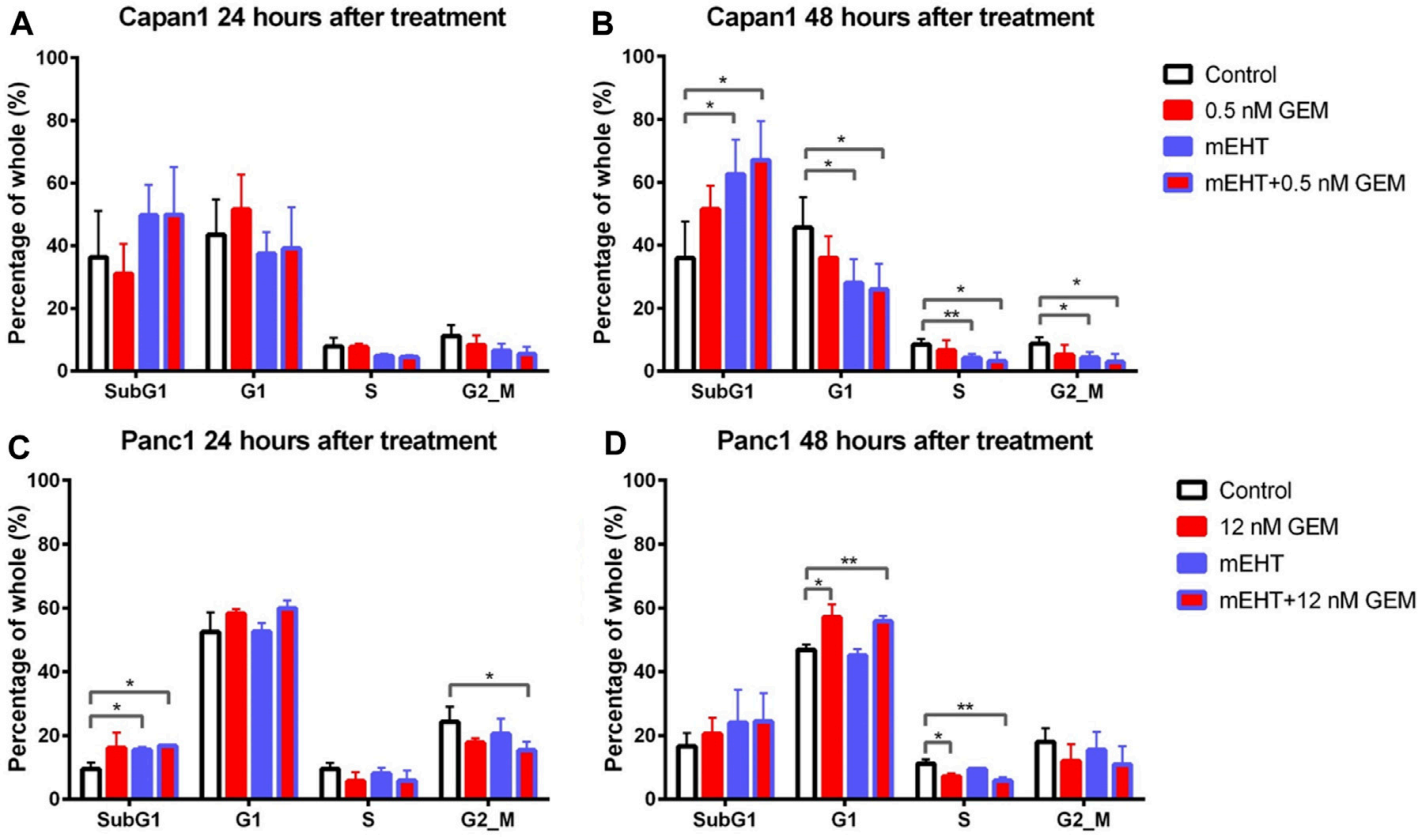
Cell cycle analysis for Capan1 and Panc1 cell lines 24 and 48 h after mEHT treatment. Capan1 cells did not show significant response after any treatment at 24 h **(A)**. Instead 48 h after treatment, a significantly elevated SubG1 phase was observed in mEHT and mEHT + 12 nM GEM groups compared to the control, followed by significantly decreased G1, S, and G2/M phases **(B)**. Panc1 cells showed an elevated SubG1 phase 24 h after treatment in mEHT and mEHT + 12 nM GEM treated groups compared to the control: *p* = 0.02 and *p* = 0.04 respectively with a decrease of G2/M phase in case of mEHT + 12 nM compared to the control (*p* = 0.04) **(C)**. At 48 h a G1 block with consecutive S phase decrease was observed in GEM mono- and combined therapy when compared to control **(D)**. (*p < 0.05 and **p < 0.01 respectively).

Though SubG1 phase apoptotic fraction did not show a major change in Capan1 cells 24 h after treatment or in Panc1 cells 48 h after treatments (Figures 5A,D), its significant elevation correlated well with the results of the Annexin V-PI apoptosis test both after mEHT and mEHT + GEM treatments compared to the control in Panc1 at 24 h and in Capan1 at 48 h (Figures 5B,C).

These results were in line with the significantly reduced G1, S, and G2/M phase cell fractions in Capan1 cells 48 h after both the mEHT and the mEHT + 0.5 nM GEM treatments, and with significantly fewer S phase in Panc1 cells in 12 nM GEM and mEHT + 12 nM GEM-treated groups 48 h after treatment (Figures 5B,D).

### Treatment-Related Upregulation of p21^waf1^ Cyclin-Dependent Kinase Inhibitor

**FIGURE 6:**
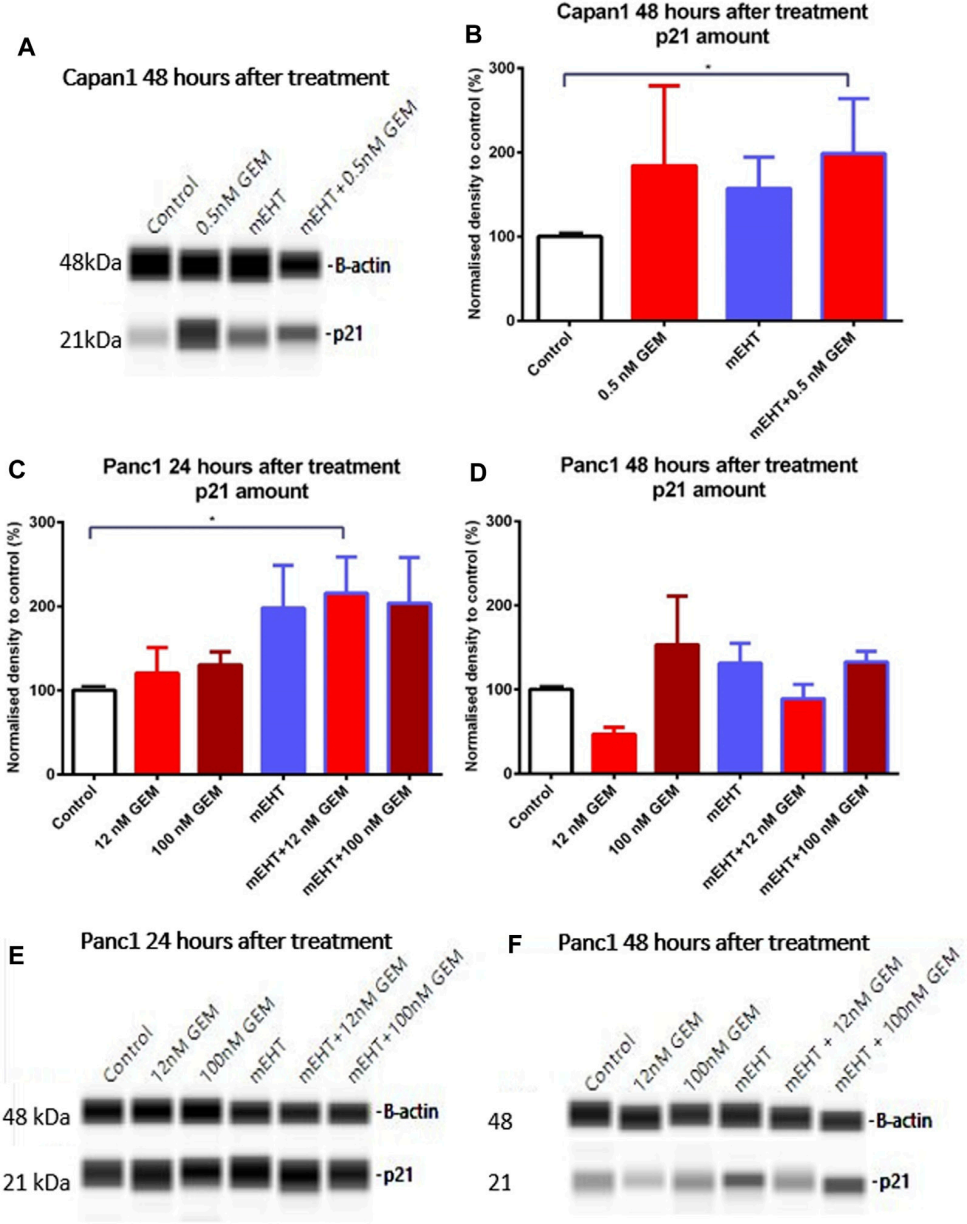
Western blot analysis of p21^waf1^ protein in Capan1 and Panc1 cells. The level of p21^waf1^ increased significantly after mEHT + 0.5 nM GEM treatment in Capan1 cells at 48 h after treatment compared to the control, as seen on the representative WES protein loading **(A,B)**. In Panc1 cells, the level of p21^waf1^ increased significantly at 24 h after treatment in mEHT + 12 nM GEM group compared to the control, *p* = 0.023, seen on the WES protein loading gel, with normalization to β-actin **(C,E)**. At 48 h the Kruskal-Wallis test showed a significant difference between the groups (*p* = 0.028) with elevated levels of p21^waf1^ in mEHT and 100 nM GEM monotherapies and in the mEHT + GEM groups, shown on representative WES protein loading images **(D,F)**.

None of the treatments changed the level of the cell cycle promoters CDK4 and Cyclin A measured by Wes Simple analysis (data not shown). However, the significant elevation of the cyclin-dependent kinase inhibitor p21^waf1^ protein levels was observed in Capan1 cells, 48 h after GEM and the combined mEHT + 0.5 nM GEM treatments compared to the controls (*p* = 0.03) (Figures 6A,B). Increased p21^waf1^ expression was also detected in Panc1 cells 24 h after combined mEHT + 12 nM GEM treatment (Figures 6C,E), which declined by 48 h after the same treatment (Figures 6D,F).

### Treatment-Related Elevated Heat Shock Protein Expression

**FIGURE 7:**
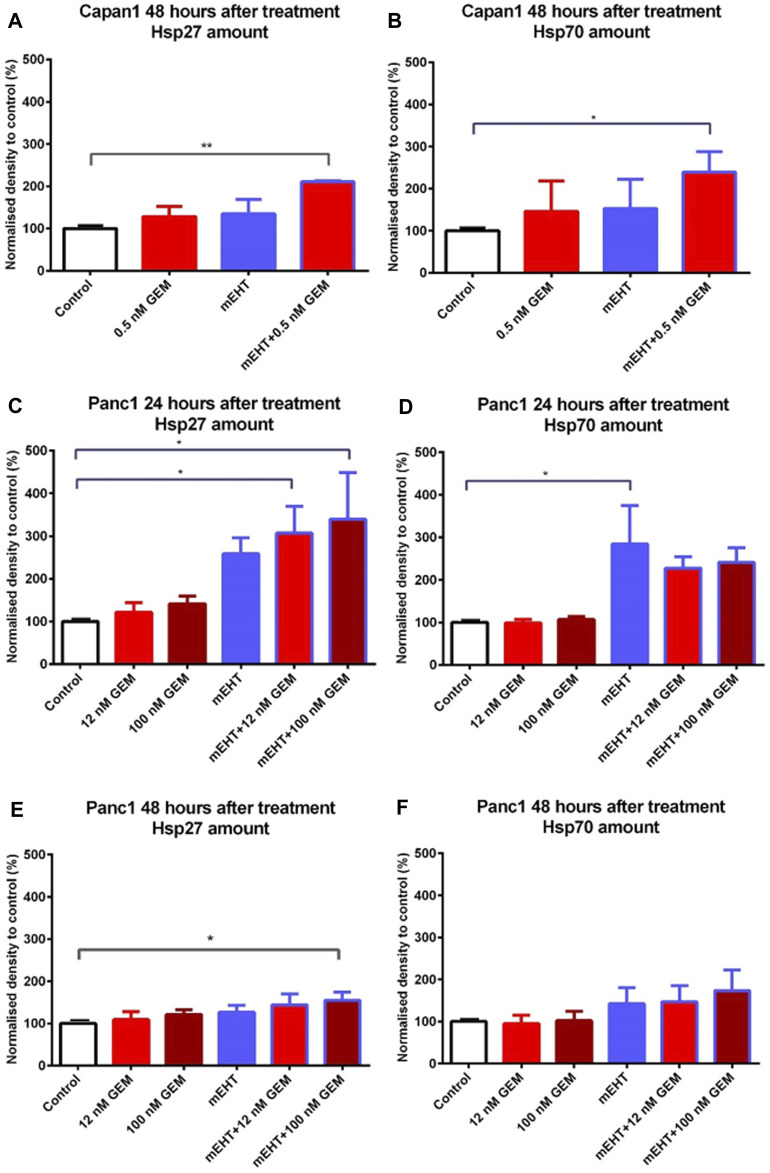
Western blot analysis of heat shock proteins. In case of Capan1 cells, the level of Hsp27 and Hsp70 proteins increased significantly 48 h after mEHT + 0.5 nM GEM treatment (*p* < 0.01 and *p* = 0.036 respectively) **(A,B)**. Hsp27 showed significantly elevated level in case of Panc1 cells 24 h after mEHT + 12 nM GEM and mEHT + 100 nM GEM treatments (*p* = 0.046 and *p* = 0.023 respectively) **(C)**. mEHT + 100 nM GEM treatment also caused significant elevation of Hsp27, 48 h after treatment (*p* = 0.014) **(E)** Panc1 cells showed elevated Hsp70 level at 24 h posttreatment only after mEHT compared to control (*p* = 0.046) **(D)**. The elevation of Hsp70 disappeared at 48 h, but none of the treatments enhanced Hsp70 protein levels significantly **(F)**.

To analyze the treatment-associated cell stress, we looked for changes in heat shock proteins including Hsp27, Hsp70, and Hsp90. The combined mEHT + 0.5 nM GEM treatment of Capan1 cells elevated both in Hsp27 and Hsp70 levels after 48 h (Figures 7A,B). In Panc1 cells, mEHT + GEM treatments induced a significant increase of Hsp27 expression (Figure 7C), while Hsp70 levels showed a major increase after mEHT monotherapy only (Figure 7D) 24 h after treatment. In Panc1 cultures 48 h after treatments, only the mEHT + 100 nM GEM treatment caused significant increase in Hsp27 levels (Figure 7E), while Hsp70 expression did not reach significance (Figure 7F). Hsp90 level did not show significant elevation either in Capan1 or in Panc1 cells after any treatment applied (data not shown).

### Treatment-Related Effects on Calcium-Dependent Cell-Cell Adhesion

**FIGURE 8:**
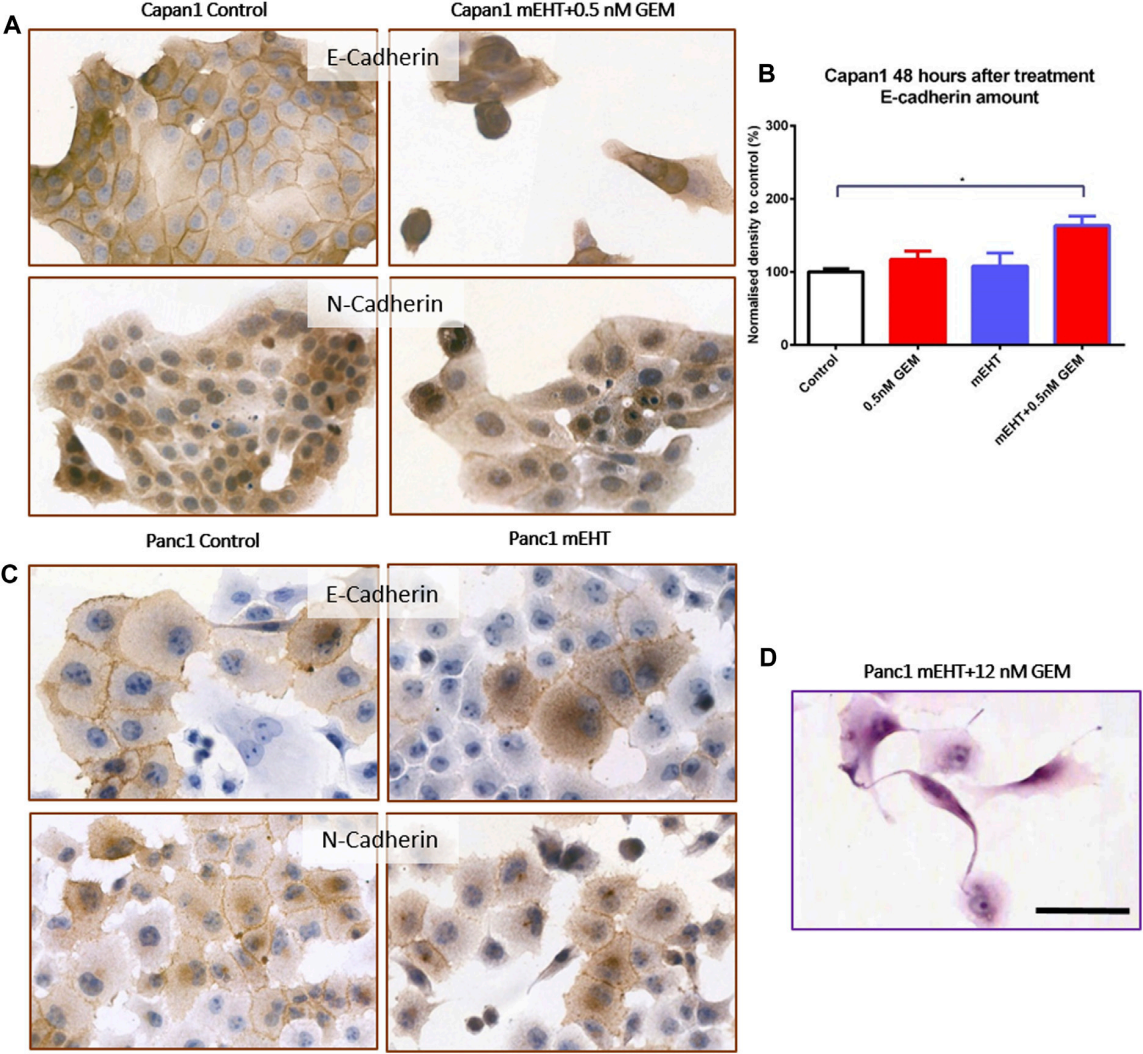
Western blot and immunocytochemistry analysis of cell membrane adhesion molecules. E-Cadherin level in Capan1 cells increased significantly 48 h after mEHT + 0.5 nM GEM treatment compared to the control (*p* = 0.018) **(B)** which manifested in decreased membrane localization of the proteins **(A)**. In case of Panc1 cells, the immunocytochemistry showed the presence of E-Cadherin and N-Cadherin in the cell membrane but no major difference in their amounts was detected after mEHT treatment **(C)**. Representative morphological deformation of Panc1 cells after treatment on hematoxyline-eosine staining **(D)**. Scale bar 50 µm.

Reduced E-Cadherin and elevated N-Cadherin calcium-dependent cell adhesion molecule levels may be linked to increased migration, epithelial mesenchymal transition (EMT), and metastatic potential. Cell-cell adhesion between Panc1 cells is strong therefore some damaged apoptotic cells grab one of their neighbors and form long processes between them (Figure 8D). E-Cadherin expression significantly increased 48 h after mEHT + 0.5 nM GEM treatment in Capan1 cells, and N-Cadherin levels also showed a minor elevation (data not shown). However, none of the other treatments affected Cadherin levels in any of the cells or time points (Figure 8).

### Treatment-Induced Inhibition of Tumor Progenitor Colonies

**FIGURE 9:**
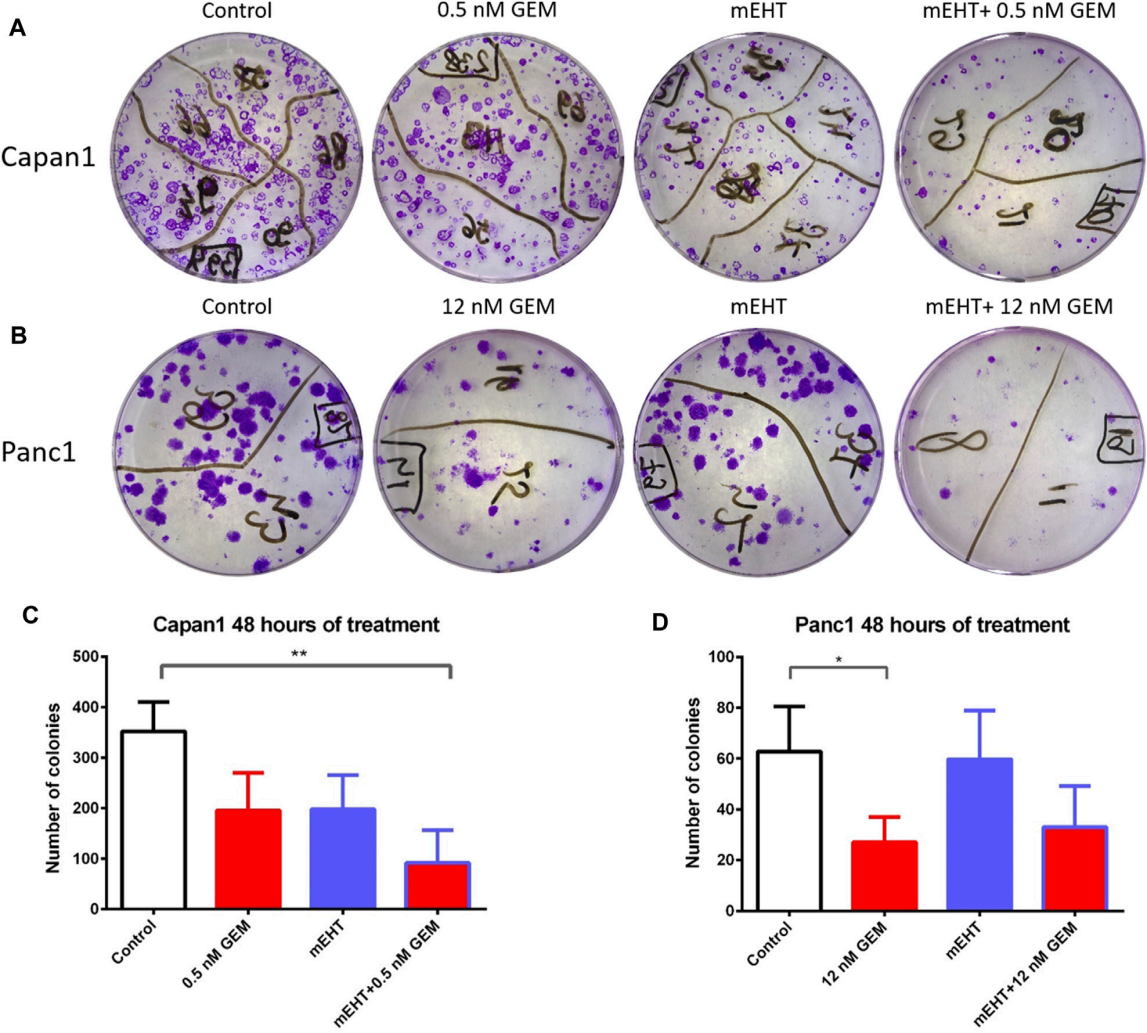
Representative images of colony forming assays of Capan1 and Panc1 cells stained with crystal violet, at the day 14 **(A,B)**. Statistical analysis of colony forming assay showed a significant decrease in the number of Capan1 colonies in mEHT + 0.5 nM GEM group compared to the control (*p* = 0.0042). Number of Panc1 colonies also reduced after 48 h 12 nM GEM treatment (*p* = 0.04) (**C,D)**.

Therapy resistance could be assessed by the survival of tumor progenitor/stem cells, therefore, we analyzed the efficiency of treatments on tumor progenitors using colony formation assay (Figures 9A,B). 48 h after mEHT treatment combined with 0.5 nM GEM, reduced number of Capan1 cell colonies were detected (*p* = 0.0042) compared to the controls (Figure 9C). In Panc1 cells, the same treatment resulted in only a strong tendency of colony inhibition, and only the 12 nM GEM monotherapy caused significant reduction in colony formation (*p* = 0.04).

## Discussion

Despite the recent improvements in oncological chemo-, radio- or immunotherapies, PDACs still represent one of the most fatal malignant tumors [25]. The most frequently used chemotherapeutic agent is gemcitabine, especially for patients with poor general conditions [26]. As a noninvasive, local hyperthermia approach, mEHT is used as a complementary therapy to support the efficiency and moderate the side effects of chemo- and radiotherapies of aggressive solid tumors [27, 28]. In line with our previous study, where mEHT improved the efficacy of radiotherapy by resolving the radioresistance of Panc1 cells [22], here we found that mEHT can also be used to support gemcitabine chemotherapy in PDAC cells. In the GEM-sensitive Capan1 cells, mEHT alone induced apoptosis and elevated cleaved Caspase-3 levels, which effects were higher when mEHT and GEM was used in combination. The increased SubG1 phase tumor cell fraction was accompanied by the upregulation of p21^waf1^ and reduction of G1, S, and G2/M phase cells. The GEM-resistant Panc1 cell line also showed signs of apoptosis and cell cycle block after 24 h both in the mEHT and in the mEHT + GEM-treated groups, which effects, however, were vanished by 48 h. The cell stress also induced elevated levels of Hsp27 and Hsp70 in both cell lines, particularly after 24 h. No EMT-associated changes were observed in the expression of N-Cadherin or E-Cadherin, except in Capan1 cells where some increase in E-Cadherin was seen after combined treatment.

Based on pilot studies, we have chosen LD20 gemcitabine concentration, which proved to be 200-times higher for Panc1 than for Capan1, which is in line with the higher GEM resistance of Panc1 cell line [29]. Similar to our earlier published results [22], a 60-min mEHT-treatment induced significant apoptosis in Panc1 cells after 24 h, which however, diminished by 48 h. Also, a significant cell death response was observed in Capan1 cells both after 24 and 48 h, which effect was increased further when mEHT was complemented with 0.5 nM GEM.

All effective treatment modules resulted in cell loss and morphological signs of apoptosis as we observed earlier [22]. These included chromatin condensation, nuclear shrinkage, the formation of apoptotic bodies, and cell membrane blebbing. In addition, elongated damaged cells attaching to distant neighbors were also observed due to the strong adherent mechanisms in both cell lines. Apoptosis as the main mechanism behind treatment-related cell damage was detected in both cell lines after Annexin V/PI double-staining. Capan1 cells behaved similarly to SW1990 PDAC GEM-sensitive cell line when mEHT was applied resulted in a progressive loss of viable cells and the apoptotic ratio reached 50% 48 h after mEHT + GEM treatment [30]. Even after efficient treatments with mEHT or mEHT + GEM, a less apparent programmed cell death response was seen in the GEM-resistant Panc1 cells. This is in line with a previous study about Panc1 and BxPC3 PDAC cells where combination of magnetic hyperthermia and gemcitabine was used and in which only discreet apoptotic cell death response was found after monotherapies. Similar to us, they also observed the cumulative effect of hyperthermia + gemcitabine [31]. This supports the potential sensitization effect of mEHT in GEM-resistant PDAC cells when monotherapies fail. Apoptosis as the dominant type of programmed cell death response to mEHT has been confirmed in several tumor types. This can be induced *via* both the extrinsic pathway through activation of Caspase-8 and the mitochondrial damage response, finally resulting in the cleavage and activation of Caspase-3 [17]. Here we detected the mitochondrial to cytoplasmic release of cytochrome C, which is a key event in the downstream apoptosome formation and Caspase-3 activation [32]. Indeed, we also demonstrated an elevated number of damaged tumor cells with nuclear cleaved Caspase-3 positivity in both Capan1 and Panc1 cells after treatments, and the upregulation of BAX protein in mEHT treated Panc1 cells. Similar mechanisms were also revealed in Panc1 when mEHT was combined with radiotherapy [22], and after mEHT in melanoma and lung cell lines [33, 34].

Our results are also consistent with those results which revealed elevated apoptosis and reduced G2/M phase tumor cell fractions after mEHT + GEM treatment in SW1990 PDAC cell line, which also carries multiple cancer–related driver mutations [30]. The same G2/M and S phase block were presented in lung adenocarcinoma cells after 3 h of conventional hyperthermia [35]. We observed decreased G2/M and S phase cell fractions in case of both Capan1 and on Panc1 cells after combined mEHT + GEM treatment. Interestingly, the G1 phase was elevated when hyperthermia and GEM treatment were applied concomitantly in BZR-T33 lung cancer cells and in Panc1 cells but it decreased in PL5 and Capan1 PDAC cells [36]. In the present study, the cyclin-dependent kinase inhibitor p21^waf^ protein was also upregulated after combined treatments but without the notable reduction of the cell cycle promoters CDK4 or CyclinA. These strengthen our previous results related to the role of elevated p21^waf1^ levels after mEHT both in CRC cells and in PDAC [19, 22]. A similar effect induced by microwave hyperthermia in non-small cell lung cancer [37] was observed, along with elevated Chk2 levels, which can also explain G2/M phase depression.

Hsp27 has been described as a predictive and prognostic factor in PDAC cases [38]. Cell lines overexpressing Hsp27 were observed to show increased potential for apoptosis and cell cycle block with S phase depletion after conventional hyperthermia [36]. Induced Hsp27 overexpression sensitized tumor cells to gemcitabine after 1–1.5 h water bath heating, as opposed to Capan1 and Panc1 cells, this was the reason why other PDAC cell lines were also tested. Here we used an advanced hyperthermia device which could induce a 3-fold elevation of Hsp27 in Panc1 cells 24 h after mEHT treatment and when this was combined with GEM an even higher elevation of Hsp27 and Hsp70 was observed. However, a statistically non-significant, but still remarkable 1.5-fold Hsp27 elevation was observed both in Capan1 and in Panc1 48 h after mEHT monotherapy, when Hsp70 and Hsp90 levels showed only small mEHT-induced increase. Recently, *in vivo* mEHT monotherapy of triple-negative breast cancer, revealed a direct apoptotic effect and the exhaustion of Hsp70 [39].

Metastatic potential is one of the main reason for the poor prognosis of PDAC [40]. Both Capan1 and Panc1 cells showed an inherently high coherence and membrane expression of Ca^2+^-dependent homophilic adhesion-mediated through E-Cadherin [41]. In our study, the E-cadherin levels partly translocated to the cytoplasm, increased in mEHT + GEM treated cells, which might indicate a reduced migratory ability of tumor cells. N-Cadherin was also expressed in both cell lines, suggesting that they have some inherent potential to EMT (epithelial mesenchymal transition), which, however, did not show a noticeable change after any treatment. Though reduced migration of Panc1 cells was published after combined mEHT and GEM treatments in a highly GEM-resistant *in vitro* model [42] but they used a 50× higher dose of GEM for LD50. Treatment resistance can be mostly linked to the survival of tumor stem/progenitor cells [43]. We found significant reduction of colonies in Capan1 cultures both after mEHT and 0.5 nM GEM treatments, which effect became additive in mEHT + GEM group. The GEM-resistant Panc1 cells also showed a decrease in colony formation after combined treatment.

## Conclusion

In conclusion, mEHT could induce cell viability loss, apoptotic cell death, and cell cycle block on the Capan1 PDAC cell line. When mEHT was followed by GEM, cell death induction was also observed in GEM-resistant Panc1 cells. The major pathway of apoptotic cell death was caspase-dependent which was confirmed by the increase in Caspase-8 positive cells and BAX levels the mitochondrial release of Cytochrome C and activation of Caspase-3. These were accompanied by the upregulation of p21^waf1^ protein and the decrease of S and G2/M phase cell fractions. In both cell lines, Hsp27 levels were induced after combination treatments, along with the reduction of the resistance-related tumor progenitor cell colonies and increased expression of the cell adhesion molecule E-Cadherin in Capan1 cells. Therefore, our results suggest a role of mEHT in sensitizing PDAC cells to an efficient GEM treatment.

## Data Availability

The raw data supporting the conclusions of this article will be made available by the authors, without undue reservation.
